# Exploration of clinical ethics consultation in Uganda: a case study of Uganda Cancer Institute

**DOI:** 10.1186/s12910-024-01085-1

**Published:** 2024-08-09

**Authors:** Mayi Mayega Nanyonga, Paul Kutyabami, Olivia Kituuka, Nelson K. Sewankambo

**Affiliations:** 1 College of Health Sciences, School of Medicine, Department of Anatomy, Makerere University, Kampala, Uganda; 2 Joint Clinical Research Center, Lubowa, Kampala Uganda; 3 College of Health Sciences, School of Health Sciences, Department of Pharmacy, Makerere University, Kampala, Uganda; 4 College of Health Sciences, School of Medicine, Department of Surgery, Makerere University, Kampala, Uganda; 5 College of Health Sciences, School of Medicine, Department of Medicine, Makerere University, Kampala, Uganda

**Keywords:** Ethical dilemmas, Ethical issues, Clinical ethics consultation, Clinical ethics support services, Uganda

## Abstract

**Introduction:**

Globally, healthcare providers (HCPs), hospital administrators, patients and their caretakers are increasingly confronted with complex moral, social, cultural, ethical, and legal dilemmas during clinical care. In high-income countries (HICs), formal and informal clinical ethics support services (CESSs) have been used to resolve bioethical conflicts among HCPs, patients, and their families. There is limited evidence about mechanisms used to resolve these issues as well as experiences and perspectives of the stakeholders that utilize them in most African countries including Uganda.

**Methods:**

This phenomenological qualitative study utilized in-depth interviews (IDIs) and focus group discussions (FGDs) to collect data from Uganda Cancer Institute (UCI) staff, patients, and caretakers who were purposively selected. Data was analyzed deductively and inductively yielding themes and sub-themes that were used to develop a codebook.

**Results:**

The study revealed there was no formal committee or mechanism dedicated to resolving ethical dilemmas at the UCI. Instead, ethical dilemmas were addressed in six forums: individual consultations, tumor board meetings, morbidity and mortality meetings (MMMs), core management meetings, rewards and sanctions committee meetings, and clinical departmental meetings. Participants expressed apprehension regarding the efficacy of these fora due to their non-ethics related agendas as well as members lacking training in medical ethics and the necessary experience to effectively resolve ethical dilemmas.

**Conclusion:**

The fora employed at the UCI to address ethical dilemmas were implicit, involving decisions made through various structures without the guidance of personnel well-versed in medical or clinical ethics. There was a strong recommendation from participants to establish a multidisciplinary clinical ethics committee comprising members who are trained, skilled, and experienced in medical and clinical ethics.

**Supplementary Information:**

The online version contains supplementary material available at 10.1186/s12910-024-01085-1.

## Introduction

Globally, health care providers (HCPs), hospital administrators, patients and their caretakers are confronted with complex moral, ethical and legal dilemmas during clinical care that call for mechanisms of resolution [1–3]. Ethical challenges in cancer care have moved beyond the HCP’s competences to salient individual, cultural, economic, political, religious, and social challenges [4]. They include vagueness in informed consent, surrogate decision making, conflict in interpersonal relationships, rationing of limited resources, medical futility in intensive care units, diverse cultural interpretations of treatment, truth-telling, end-of-life decisions, and refusal of treatment. These dilemmas have led to moral distress, burnout, defensive medicine, HCPs’ job dissatisfaction, unsatisfactory patient care and reputational damage to HCPs and institutions [5].

There has been exponential growth in the application of ethical and moral judgments in addressing medico-ethical issues at the hospital bedside [6–8]. Ethical decision making in bioethics predates the 1960s with classical examples of the *God’s committee* where judgements on who to live or die in the context of scarce resources were made [9]. To date, similar questions continue to challenge governments, bioethicists, clinical ethicists, HCPs, patients, and their families [10, 11].

In high-income countries (HICs), both formal and informal clinical ethics support services (CESSs) are employed to address bioethical conflicts among HCPs, patients, and their families [12, 13]. Well established CESS mechanisms include clinical ethics committees (CECs) and forums for moral deliberations such as ethics reflection groups and ethics rounds [14, 15]. The characteristics, functionalities and effectiveness of these mechanisms have been well documented [16–18]. However, in Africa, where medical, socio-economic, legal, and cultural complexities abound, a variety of ethical dilemmas are equally pronounced [4], with scanty evidence of approaches for their resolution. Limitations to formation and access to CESSs include inadequate knowledge of these mechanisms and processes, misconceptions about ethical consultations by patients and HCPs, power imbalances between HCPs and patients or their families, lack of time, inadequate qualified ethicists as well as limited resources to establish formal ethics consultations [19, 20].

In Uganda, patient care grapples with resource scarcity, high disease burden, poor health-seeking behaviours and adherence, emotional and psychosocial factors, communication gaps, limited knowledge, and slow medical technology progress. Notably, challenges for cancer patients such as transition to end-of-life, and honouring patient choices are complex and come with moral dilemmas for HCPs, patients, and their caregivers in addition to causing distress among HCPs [21].

The Uganda Cancer Institute (UCI), treating patients from diverse backgrounds and utilizing advanced therapies, inevitably encounters complex ethical issues. Understanding the current CESS offerings at UCI is crucial for improvement. This study explored the approaches utilized to resolve ethical dilemmas at UCI.

## Methods

### Study design

To address this knowledge gap at the UCI, we conducted an exploratory qualitative study using a phenomenological approach, employing in-depth interviews (IDIs) and focus group discussions (FGDs) as data collection methods.

### Study setting

Established in 1965 by Makerere University in Uganda and the National Cancer Institute (NCI) in the USA, the UCI is a renowned East African centre of excellence in clinical oncology care [22]. The UCI also plays a crucial role as an oncology research and training facility. Handed over to the Uganda Ministry of Health, UCI now serves as a pivotal hub for cancer care. With a bed capacity of 80, it attends to approximately 200 outpatients daily, including those from Uganda, the Democratic Republic of Congo, South Sudan, and neighbouring regions [23]. UCI provides comprehensive clinical oncology care, encompassing paediatrics, gynaecology, radiotherapy, surgery, and pharmacy services. Integrated within these disciplines are palliative care, counselling, and social support services. Despite its crucial role, UCI faces understaffing, with a doctor-patient and nurse-patient ratio of 1:100 and 1:50 respectively [24].

With a nearly 60-year history, UCI’s autonomy and it being the only comprehensive cancer care facility in Uganda made it an ideal site for this study.

### Study participants

Participants, including UCI management and clinical staff, patients, and their caretakers (aged ≥ 18), present at the UCI during the study, were purposively selected. Eligible individuals spoke English or Luganda, had previously faced challenging issues in their care, and were willing to undergo audio-recorded interviews. To identify potential participants meeting the inclusion criteria, the head of research, social workers, counselors and patient advocacy groups were contacted, and a compiled list of these individuals was then created. Potential participants were reached out to via email, SMS and phone call for the interview appointment. Only participants who provided written informed consent were recruited into the study.

### Data collection methods and instruments

Data was collected qualitatively using IDIs and FGDs in December 2023. Interview questions were guided by a semi-structured interview guide that was developed by the authors who followed a reflexive, iterative, and dialogic processes, that directly addressed the research inquiry. The focus of the guide included understanding definitions of clinical ethics consultations, commonly experienced ethical issues, mechanisms for resolution, existence of CESSs as well as recommendations to improve clinical ethics consultations. Interviews, conducted with the participants’ informed consent, were moderated by MMN and a hired professional research assistant who took notes.

Two interview guides, one for HCPs and another for patients and their caretakers were created and tested with 2 HCPs, 2 patients, and 1 caretaker to ensure the guides’ validity and reliability. The final guides were then refined to address any repetition or incomplete information. The updated interview guides were used to investigate the mechanisms employed in resolving ethical dilemmas at the UCI, factors that influenced these consultations and perspectives and experiences of stakeholders that utilized these services. Initially, the questions were formulated in English and subsequently translated into Luganda by a certified translator at the Makerere University, Department of African Languages, who had a comprehensive understanding of both languages. The Luganda translations were reverse translated into English to verify the accuracy of the translated versions. Luganda was a preferred language for translation because it is a commonly understood local language.

All interviews were conducted within UCI in respective offices of the management and clinical staff, and for patients and caretakers, within a secured study room.

Interviews lasted between 45 min and 1 h, were recorded using a digital recorder and thereafter transcribed and anonymized. Each day, Luganda audio files were translated to English by a certified translator at Makerere University. These translated files were then transcribed by the principal researcher, MMN. Data collection was guided by the principle of saturation, a point at which no new themes emerged from interviews and data collection was suspended [25, 26].

### Data analysis

Audio recorded interviews were transcribed verbatim. The transcriptions were then prepared and entered into Nvivo 12 qualitative scientific software for analysis. The COREQ checklist (Supplementary file 2) was applied to ensure adherence to criteria for qualitative research [27]. The data underwent thematic analysis [28], and both inductive and deductive approaches to qualitative data coding and analysis were employed. With reference to the interview guide and objectives, deductive analysis involved the development of a predefined coding framework. In contrast, inductive analysis included the creation of additional codes during the transcript review which expanded the codebook. MMN and a research assistant coded the data. All authors then discussed and refined the themes until reaching consensus. Through in-depth examination of transcripts and field notes, the authors collaboratively derived meaning and interpretation from the data. New codes were generated to address emerging areas of inquiry not initially covered in the codebook. Finally, the data was indexed, charted, and interpreted by all authors.

For purposes of validating and verifying findings, data was triangulated through taking notes with a research assistant that was experienced in qualitative research. No inconsistencies were identified in the data sets during transcription. Member checking was performed on 4 IDI participants (3 HCPs and 1 patient). Transcripts for these 4 participants were shared with them for review and they all confirmed the accuracy of information transcribed.

### Ethical considerations

This research was reviewed and approved by the Makerere University School of Biomedical Sciences Research and Ethics Committee. Administrative clearance was also obtained from UCI to conduct the study there.

Participant information was kept confidential with identifying information (such as name) encrypted and stored separately from any study data. Only the authors and the research assistant had access to the password protected dataset. Informed consent forms, recruitment materials and interview notes were stored in lockable cabins, under lock and key, with only restricted access to the research team.

This was a minimal risk study whose inconvenience to participants were some discomforting sensitive information, sociodemographic, clinical, and behavioral questions. Participants were free not to take part or to discontinue participation in the study at any time. Potential participants were informed that refusal to participate or withdrawal from the study would not affect a patient’s treatment plan nor affect the staff’s employment (in the case of HCPs) at UCI. The participant’s decision to participate or not was kept confidential.

## Results

With 100% response rate of participants, 21 IDIs were conducted: 12 with UCI Staff (5 female and 7 male) and 9 with patients (5 female and 4 male). Additionally, three FDGs were held: two with patients and one with caretakers. Each patient FGD consisted of 6 participants. One patient FGD had female participants while the other had only male patients. The caretaker FGD included 10 mixed-gender participants (6 female and 4 male).

All IDIs with UCI staff participants were conducted in English. For patient participants, 5 IDIs were conducted in English, and 4 in Luganda. One patient FGD was conducted in English, and the other in Luganda. The FGD with caretakers was conducted in Luganda.

All UCI staff participants had at least attained tertiary education (see Table 1), with the highest level of education being post-doctorate level. However, no training specifically focused on bioethics or clinical ethics.

**Table 1:** Participant demographics

Population Demographics	Number of patient participants	Number of caretaker participants	Number of UCI staff participants
**Age (years)**			
20–30	6	1	-
31–40	10	5	5
41–50	5	4	5
51–60	-	-	2
***Gender***			
Male	10	4	7
Female	11	6	5
***Education status***			
No formal Education	8	2	-
Primary education	5	3	-
Secondary education	4	2	-
Tertiary	4	3	12
***Years of receiving*** ***care/ work at UCI***			
1–5	16	7	2
5–10	5	3	6
11–20	-	-	4

Based on the analysis, three main themes and two sub-themes were identified. The first main theme was mechanisms for resolution of ethical dilemmas with sub-themes as ethical issues/ dilemmas, and existing measures/policies to guide resolution of ethical dilemmas. The second main theme was factors influencing clinical ethics consultation. The third was strategies and recommendations for improving clinical ethics consultation.

Highly intricate, demanding, and significant ethical quandaries were prevalent among patients, caretakers and HCPs (see Table 2).

### Main theme 1: mechanisms for resolution of ethical dilemmas

Many patients and caretakers were unaware of existing mechanisms utilized to resolve ethical dilemmas.

*“Honestly*,* for the time I have spent in the hospital*,* I am not aware of any mechanism put in place to address such issues. I do not think there are formal systems or structures for solving dilemmas.” (FGD 3*,* respondent 4)*.

*“I do not know of any. But I think there should be protocols somewhere.” (IDI-13)*.

There were also patients who feared to speak up and thus represent a group whose ethical dilemmas do not get addressed.

Management and clinical staff participants reported six meeting forums used for resolving ethical dilemmas as summarized below. However, they did not provide detailed information on how resolutions were reached.

#### Individual consultation

Some patients and their caretaker consulted with counsellors, social workers, or their doctors, who helped them resolve ethical issues at the individual level.

*“At the beginning*,* I had fears of removing my womb but my doctor told me it was a health risk to me and he explained why they’re doing it. He ironed out the issue very well and I started to reason with him…”(IDI-06)*.

*“Me*,* I’m an open person*,* I always go to the senior doctor and tell him what is hurting me. The senior doctor always helps me to get a solution…” (IDI-07)*.

#### Tumor Board meetings

Some ethical conundrums were discussed in tumor board meetings where complex cancer cases were discussed.

*“We use the tumor boards. These tumor boards involve many disciplines; medical oncologists*,* radiation oncologists*,* nurses*,* pharmacists*,* radiologists*,* pathologists. So there when patients are discussed*,* an appropriate treatment plan is decided on by the team. And in the department*,* like in radiotherapy*,* we have Thursday departmental meetings*,* where we discuss patients before they start treatment.” (IDI-18)*.

#### Morbidity and mortality meetings (MMMs)

Some ethical issues were reported to be discussed during MMMs. With an aim of improving service delivery, any social and ethical issues that might have impacted treatment plans were addressed in this forum with strategies to mitigate them from recurring.

*“…what could have caused the death of the patient? was the death avoidable? is the cause attributed to negligence. Such issues can still be addressed by the morbidity and mortality manager at morbidity and mortality meetings…” (IDI-10)*.

#### Rewards and sanctions committee meeting

Previously known as the *disciplinary committee*, the rewards and sanctions committee was believed to handle some ethical issues and dilemmas as part of the disciplinary inquiries.

*“The Rewards and Sanctions committee is composed of 5 people. It used to be called the disciplinary committee in the old days. We thought it would be good to motivate the staff that performed well at work*,* so we changed the name. Individuals with complaints forward them to that committee and then the committee goes through them and decides what to do.” (IDI-18)*.

#### Clinical departmental meetings

Were also utilized by HCPs to address ethical conundrums alongside discussing challenges faced within different clinical departments.

*“Team meetings*,* or departmental team meeting handle such scenarios and decide what to do…” (IDI-15)*.

#### Core management meetings

Were weekly leadership meetings that were also reported to have been an opportunity for different UCI staff to share any challenges of complex decision making during clinical care.

*“….Issues can also be addressed by the UCI core management. So whatever issue that comes up*,* depending on the level of its magnitude*,* it can be addressed by whatever level of managers that we have.” (IDI-10)*.

### Subtheme 1: ethical issues/dilemmas encountered by HCPs, patients, and caretakers

Ethical issues that were reported to be resolved by the mechanisms explored included paternalism, informed consent, privacy, and confidentiality.

#### Paternalism

A considerable number of patients and their caregivers held the belief that their physicians possessed comprehensive knowledge and depended on their expertise and experience to provide sufficient care.

*“The doctor knows better and has experience about the treatment I am receiving so I cannot object to what he decides. Even if I am feeling so weak and the doctor says I have to continue with the chemotherapy*,* I continue because I am not the doctor…” (IDI-07)*.

#### Privacy and confidentiality

On observation, the UCI had a significant number of patients but limited space available for triage during examination. The high patient volume made it impractical to assess individuals in an environment that guaranteed privacy.

#### Informed consent

There was expression of inadequately sought informed consent. One participant reported discomfort during the discussion of her case at an expert forum. She was taken aback by the number of people present at the meeting to discuss her case.

*“They took me to tumor board to discuss my breast cancer issue. They made me remove my blouse and expose my breast as the team discussed about it. I felt so uncomfortable*,* but I had nothing to do. I need help. All I want is to be fine.” Started crying… (FGD-02 – Respondent 4)*.

Staff, patients, and caregivers at the UCI encountered challenging scenarios where decision-making appeared complex. Challenges included conflicting beliefs and values influenced by religion, culture, and interpersonal relationships, complexities in deciding for minors, issues related to benevolent deception, treatment choices based on financial considerations, power imbalances, healthcare resource rationing, and conflicts of interest.

#### Conflicting beliefs and values

Numerous patients held religious and cultural beliefs that diverged from the conventional cancer treatment options endorsed by doctors, leading to complexities in decision-making. Beyond socioeconomic factors, decisions regarding care were sometimes influenced by the perspectives of friends, family, and other patients within the cancer community. Some individuals mentioned using traditional herbal remedies alongside chemotherapy, posing a challenge for HCPs in selecting appropriate treatment options. HCPs reported concerns that herbal medicines could potentially interact with chemotherapy, adversely affecting their patients’ prognosis.

*“… my friends and family told me to try drinking herbal medicine and I am using them also. They gave me the number for the herbalist. I also know cancer patients can’t be healed; you just die. So*,* I don’t know what to do…” (FGD-02 – Respondent 2)*.

*“Some patients experience unprecedented side effects whilst taking their chemotherapy and upon intervening*,* you realize they are taking herbs. These patients are desperate and listen to false testimonials of herbalists that claim to cure cancer. As a doctor*,* I really don’t know how to help such a patient because I believe these herbalists put chemotherapy in their herbs and deceive our patients” (IDI-07)*.

#### Complexities in deciding for minors

HCPs faced challenges in determining the appropriate course of action for minors whose parents made choices based on their religious and cultural beliefs. Uncooperative parents even made decision-making harder. Issues concerning competent minors without legal capacity to consent were also raised. Many physicians worried about potential legal repercussions that could adversely affect both their personal and the hospital’s reputation if they treated these children. They also expressed reservations about Uganda’s lengthy legal processes, which were deemed time-consuming and had the potential to interfere with their daily patients care duties.

*“A child of 16 years comes in alone to get their chemotherapy*,* but they do not get it. You know why? Because she is a minor with no caretaker to consent*,* yet they need chemotherapy. This chemotherapy comes with side effects*,* and these children need support from caretakers or guardians. What if this child dies*,* who is responsible? What if they ask who consented on their behalf? Tell me*,* what would you do if you were the doctor? It becomes very difficult to decide how to help this child.” (IDI-08)*.

*“Some children with solid tumors require surgery but the mother thinks the child is going to die if the surgery is done. But if you don’t do the surgery*,* the child is going to die anyway. It becomes extremely difficult to decide for such a child when their parent does not allow to the recommended care.” (IDI-04)*.

#### Resource allocation

The allocation of scarce resources during rationing posed ethical challenges at the UCI. Limited resources, technology, and supplies, were reported. During data collection, floor cases were observed on the wards. Many caretakers reported inadequacies in wheelchairs, leading them to carry their patients to observation rooms. A significant number of patients conveyed that their numbers did not allow all of them to receive timely radiotherapy, leading to disease progression and a bleak prognosis. Compounding this issue, the occasional dysfunction of radiotherapy machines meant that some patients missed their scheduled radioactive treatments, a situation also reported for patients on the surgery list. With specific days allocated for different surgeries, many patients had lost hope of their turn ever arriving.

Nurses highlighted challenges with the insufficient availability of oxygen ports, creating dilemmas about prioritizing patients in need of oxygen. They also mentioned a shortage of nursing staff relative to the high number of patients, making it impossible to attend to everyone efficiently and equally.

*“Now*,* I want you to imagine*,* you are one nurse or two working on forty patients who are critically ill. Remember*,* one patient alone can make you extremely tired*,* but now you’re having forty critically ill and don’t what to lose any life. You get confused about whom to start with. By the time you complete*,* you don’t want anybody talking to you*,* you are really tired and burnt out. Of course*,* they will say you are ignoring them but still there is no way you can run in between and suspect that may be this one wants attention*,* you won’t even know how somethings happen…”(IDI-05)*.

*“Resources are limited here*,* sometimes as much as you want to be ethical you may be constrained by limited resources …” (IDI-16)*.

*“…because of increasing number of patients*,* human resource is little. There is also not enough infrastructure*,* and the overwhelming numbers sometimes hinders privacy because patients need to be talked to one by one. Patients also don’t like to disclose their names*,* but here someone has to stand up and read the client’s name aloud…” (IDI-19)*.

#### Truth telling

Some caregivers expressed a preference for keeping their patients unaware of their cancer diagnosis, urging HCPs to administer treatment without disclosing the nature of the patient’s condition. Conversely, others were comfortable with their patients being aware of the cancer diagnosis but requested doctors to refrain from sharing all details. Many physicians observed that these situations presented challenges to their duty of veracity, or truthfulness, to their patients.

*“I do not want my patient to know everything. Sometimes when I go to the doctor’s*,* I use English because the patient does not understand English. Ha ha ha… I don’t want my patient to lose hope because he is always thinking about death and says he is ready to die. I do this to help him. Imagine if he hears that some organ has been affected by chemo*,* I would be the one to suffer. I need him to receive his treatment in peace.” (IDI-13)*.

### Subtheme 2: existing policies and measures to guide resolution of ethical dilemmas

The UCI lacked established policies specifically addressing clinical ethics consultations. Reports indicated that the development of an ethics code of conduct at UCI was underway, which would supplement the existing client charter and professional codes of conduct. These documents collectively aimed to provide guidance for HCPs in their decision-making processes.

*“…At the moment*,* there is a document which is going to come out in the next one or two months about ethical code of conduct for UCI*,* and I’m spearheading it*,* it’s almost in its terminal stages…” (IDI-18)*.

*“No*,* we don’t have any specific ethical documented guidelines. Currently*,* we base on what is clinically regarded as right or wrong.” (IDI-19)*.

**Table 2:** Summary of ethical issues/dilemmas and their resolution mechanisms

Examples of ethical issues	Resolution Mechanisms
- Paternalism ; some patients’ decision making was made by their physician	- Individual consultation
- Informed consent; Lack of comprehension in certain cases due to difficulty in translating technical terms by HCPs made informed consent invalid	- Individual consultation
- Inadequate privacy due to high patient volumes and limited space to accommodate all of them	- Clinical departmental meetings - Core management meetings
**Encountered ethical dilemmas**	**Resolution mechanisms**
- Conflicting beliefs and values driven by religion, culture and interpersonal relationships	- Individual consultation - Tumor Board meetings - Clinical departmental meetings
- Truth-telling to patients versus benevolent deception	- Tumor Board meetings - Individual consultation - Clinical departmental meetings
- Power imbalance; Some ethical dilemmas that involved heads of department (HODs) were not reported as it is the same HODs that made up the forums where these dilemmas were resolved.	- Individual consultation
- Rationing resources; Fair distribution of limited resources among a large population of cancer patients was complex for some UCI staff.	- Clinical departmental meetings - Individual consultation - Core management meetings
**Key definitions** ***Ethical issues***: *Situations expected to arise as a matter of routine in our practice*,* a great majority of them allowing for straightforward decision making*,* because the “right” answer has been made clear through clear-cut guidelines* [5]. ***Ethical dilemmas***: *A complicated situation that when two or more ethical principles*,* values*,* beliefs or standards are conflicting with each other*,* making decision making difficult* [29].	

### Main theme 2: factors influencing clinical ethics consultations at UCI

The effective resolution of ethical dilemmas at the UCI was reportedly influenced by several factors which included;

#### Lack of sufficient space to ensure privacy

Some caretakers did not think that there was dedicated space to conduct ethics consultations.

*“… I think the space is not there. You find that the some patients sleep and dress up from the same area where triage is done from. Where do you expect such discussions to be held?…”(FGD-01-Respondent 5)*.

#### Limited knowledge in medical/clinical ethics

Concerns were raised among UCI staff regarding the ethical competence of members comprising various committees. Some HCPs, patients, and caretakers expressed doubts about the suitability of existing forums to address clinical ethics dilemmas and questioned their overall effectiveness.

*“Knowledge gap remains a big challenge*,* because if people lack sufficient knowledge on medical ethics*,* on what is medically right and morally right and differentiating the two is tricky…” (IDI-10)*.

#### Time constraints for UCI staff

Many UCI staff reported the lack of time to conduct clinical ethics consultations.

*“The workload! These doctors are overwhelmed by the patient numbers. They see so many patients. Some of them must handle administrative and human resource issues too. So*,* I don’t think such people can concentrate and come up with a good structure or find a vibrant committee that they can come to or reach out to a common man in terms of emphasizing what to be done here and there. I don’t think that time is there. They may contribute to your idea but will not come by to discuss individual ethical dilemmas.” (IDI-04)*.

*“ …even if some ethical issues might arise during these meetings*,* there is no time to discuss these issues. The agendas for the meetings are even so different and ethical issues are not priority. Take an example*,* tumor board meetings are for discussing complicated cases in terms of disease not ethics. The time for tumor board is also about 2 hours and they can discuss one patient for like 35 minutes. Now*,* if the time is not even enough to discuss all proposed patients*,* where will the time to discuss ethical issues come from? These doctors do not have time*,* they have to go and see patients.” (IDI-15)*.

#### Power imbalances

It was mentioned that certain UCI personnel misused their positions, complicating the fair resolution of ethical dilemmas in which they were involved.

*“The challenge*,* aah… I tried to talk to someone*,* but as I said*,* they are some people that are like untouchable*,* some people are aware that there is nowhere you can report them*,* may be to God. You see something*,* but someone is like an elephant so just keep quiet and suffer mentally about it.” (IDI-03)*.

#### Lack of resources to compensate HCPs

Some participants mentioned that the UCI lacked allocated funds specifically for compensating staff who handle ethical dilemmas.

*“The UCI has no money. We even struggle to get drugs and radiotherapy. Personnel handling such issues need to be funded because this is Sunday to Monday job and the patients are flocking in every day.”(FGD-03- Respondent 2)*.

#### Lack of awareness

some patients and caretakers reported of not knowing where to seek guidance for the resolution of the ethical dilemmas they faced. Moreover, these participants were willing to utilize existing platforms or committees if they knew where to find them.

*“I have never reported my case anywhere because I do now know where to report. I hear there is a tumor board but you cannot go there. It is the doctors that invite you.” (IDI-12)*.

### Main theme 3: strategies and recommendations for improving clinical ethics consultation

Most study participants overwhelmingly advocated for the establishment of a dedicated multidisciplinary clinical ethics committee, trained in clinical ethics to handle ethical dilemmas. Their reservations, however, primarily revolved around concerns related to limited funding and the absence of policies to support the establishment of such a platform.

*“We need a clinic ethics committee to oversee all the clinical ethics aspects that are going on at UCI. …we need it definitely. That is one thing that is missing at UCI.” (IDI-03)*.

*“Personally*,* I feel there should be an established committee that doesn’t depend on an Mortality and Morbidity Meetings…I would rather recommend that if a clinical ethics committee is generated and empowered*,* it should be in position to handle these ethical dilemmas on a daily basis to have better outcomes.” (IDI-11)*.

Participants outlined the composition of the envisioned clinical ethics committee and the qualities deemed essential for its members. A frequently mentioned preference was for a full-time, diverse committee comprising HCPs, expert patients, clergy, and lay individuals. Additionally, participants emphasized the committee’s responsibility to formulate guidelines and policies for addressing ethical issues and dilemmas.

Furthermore, participants highlighted the importance of committee members possessing knowledge and training in medical and clinical ethics, along with a combination of soft and technical skills to effectively engage with people in a considerate and proficient manner.

*“I think a full representation would be good because at the different service points*,* different people face different ethical issues. Team radiotherapy*,* team nuclear medicine*,* the pharmacist*,* the doctor as well as having survivors or patients come on board. (IDI-10)*

*“Someone’s behavior is important. Like someone should not be short tempered. One should be calm and able to handle different people without bias or favoritism.” (FGD-02*,* Respondent 4)*.

## Discussion

This study explored the approaches utilized to resolve ethical dilemmas at Uganda’s cancer treatment, research and education center. The study also illuminates already existing worldwide ethical issues and dilemmas in health care [30–33]. The ethical issues and dilemmas experienced at the UCI ranged from the minor to the more complex ones including; paternalism, conflicting beliefs and values, benevolent deception, inadequate observance of informed consent processes, privacy and confidentiality, and severe resource rationing.

Our results revealed an overwhelming desire by HCPs, patients, and their care givers to have effective mechanisms to address these ethical issues and dilemmas. The mechanisms employed at the UCI have had some promising outcomes, but limitations remain in their suitability, application, and sustenance among others to which researchers, practitioners and policy makers can draw lessons. Six notable approaches were utilized to address these ethical quandaries depending on the patients, caretaker and HCP motivations and preferences, nature of the dilemma and availability of resources.

### Ethical issues/dilemmas

#### Paternalism

This highlighted finding is reported to be a common practice in LMICs [34]. Low literacy levels among patients and caretakers in these populations [35] have given doctors an inevitable central role to decision-making in their patients’ clinical care [36], negating the significance of patient autonomy and self determination. Adopting a patients-centered approach is crucial to ensuring a more collaborative relationship where HCPs realize rights of patients and their caretakers.

#### Conflicting beliefs and values

It is not uncommon for patients and their caretakers to seek healthcare with beliefs and values that HCPs do not deem medically appropriate [37]. These differing values and beliefs can create biases among HCPs, potentially increasing discriminatory care and undermining their obligation to support patient self-determination. HCPs have sometimes refrained from providing certain medical services on religious grounds [38, 39], further complicating the provision of care as well as undermining patients’ trust in healthcare systems. It is important that institutions and policy makers create a healthcare environment that supports patient autonomy and fosters culturally competent HCPs.

#### Benevolent deception

Our findings a preference for caretakers to withhold certain information from their patients. Virtuous traits of compassion, kindness and beneficence have been used be used to justify the moral good of lying to patients in the developed world [40]. However, truth telling is crucial to ensuring a trustworthy relationship between HCPs and patients. Communicating poor prognosis in cancer care can be particularly difficult in situations of uncertainty [41], necessitating institutions to implement clear measures that balance honesty with compassionate deception.

#### Informed consent

Our findings revealed a significant breach in the process of adequately seeking informed consent. A 2024 study by Rebecca Kampi et al. attributed the poor informed consent practices at a cancer center in Uganda to inadequate privacy and insufficient time for information disclosure [42]. This issue is not unique to developing countries where similar challenges are observed [43]. Inadequate information compromises patients’ ability to manage their own care and make shared decisions. Given its importance as an ethical issue in healthcare delivery and research, every effort must be made to ensure proper implementation of informed consent across various contexts to prevent patient dissatisfaction.

#### Resource allocation

Resource allocation in cancer care remains a global challenge at all levels of healthcare [44]. Our findings highlight rationing dilemmas that necessitate HCPs, institutions and policy makers to reassess their resource allocation responsibilities from an evidence-informed perspective. Doing so, is crucial to reducing inequities and disparities in healthcare among patient populations.

Drawing from this evidence, hospitals and populations in contexts and settings similar to the UCI should take note, and make considerations in patient management as they usually face similar ethical challenges.

#### Approaches to resolution

Patients and caretakers addressed straight forward ethical issues through one-on-one consultations with their HCPs. In contrast, complex ethical dilemmas were typically deliberated on in formal settings such as tumor board meetings, MMMs, rewards and sanctions committee meetings, core management meetings and clinical departmental meetings, all of which followed scheduled rosters. These approaches are similar to clinical ethics resolutions used in different settings globally [45, 46].

Unlike the structured clinical ethics consultation services prevalent in developed nations, fora utilized at the UCI, have been scrutinized by HCPs for their implicit and non-ethics-focused nature. Whereas they serve their intended purposes, they do not adequately resolve complex ethical issues. This is partly because HCPs find themselves having to juggle multiple roles across different meeting platforms, leaving them with insufficient dedicated time to tackle clinical ethics comprehensively. In addition, many HCPs have not received advanced clinical ethics training.

Advocacy for a formal way to resolve these ethical quandaries was widespread among participants. However, the absence of a formally established committee, such as a *clinical ethics committee*, to provide guidance on resolving ethical issues and dilemmas was notable. In this context the ethical dilemmas were informally reported and addressed with varying approaches and significance.

#### Individual level consultations

Resolution of ethical issues was advanced through intuition, education, and work experience of the different HCPs. The patients and caretakers’ motivations to report issues included trust, nature of the problem, education level, and previous experience with the HCP. There is wide consensus in the use of intuition in clinical practice and moral judgements to resolve ethical issues and dilemmas [47, 48]. Participants who utilized this approach claimed that it reduced turn around time in resolving dilemmas, increased a ‘personal touch’ and promoted flexibility and shared decision making. However, the effectiveness of individual consultations could be compromised if issues of differential authority and paternalism are not addressed. These sometimes limit disclosure, safe and equitable approaches in problem solving as the autonomy of patients to discuss their dilemmas is subdued. To this, researchers, practitioners have advanced to patient education, public involvement and engagement to empower patients and the public [49, 50]. Relatedly, case-based decision making with experience can reinforce judgement with commonly confronted dilemmas. This practice is associated with trust and judicious clinical assessments as widely applied [51]. However, this reliance on experience may pose challenges, as it may not realize that similar cases occurring in different contexts may impact outputs. Realistically one might encounter conflicting situations that demand a different course of action.

#### Tumor board meetings

This collaborative approach mirrors practices in Rwanda, Kenya, and Botswana, where similar strategies are employed in cancer settings to address complex dilemmas [46, 52–54]. Intriguingly, only a minimal number of respondents in this study experienced this collaborative approach. Notably, the tumor board meetings were not primarily convened for ethical and moral case deliberations. However, these considerations surfaced as integral components of comprehensive discussions which centered around the holistic care of patients. Evaluating tumor boards should include an integration of other medical issues alongside ethical dilemmas for their collateral utilization.

#### Mortality and morbidity meetings (MMMs)

In the United States, regular MMMs are mandatory for hospitals as part of their accreditation and maintenance process [55]. A study conducted by D.L. Clarke et al. demonstrated how these meetings yielded evidence of errors and their potential causes within the trauma care staff. This, in turn, contributed to the prevention of surgical errors and the overall improvement of patient care in the South African setting [56]. In Uganda, the 5% acceptance of autopsies from families [57] with poor hospital reports keeping [58] highlights the importance of integrating such a potential mechanism within different hospital settings. In addition, death registries can be consulted through the legal framework in the establishment of MMMs [59].

#### Rewards and sanctions committee meetings

Traditionally, the prevailing approach to human resource management involved disciplinary proceedings as a means of penalizing personnel for professional misconduct and inappropriate conduct. In Uganda, this practice is codified in the public standing orders and the Patients Charter [60, 61]. A recent initiative at UCI, spearheaded by departmental heads, is the rewards and sanction committee, designed to commend commendable conduct and penalize unacceptable professional practices. Specifically, the committee addresses violations of professional ethics and instances of malpractice reported by patients and caregivers. The aim is to ensure justice for patients and encourage them to report such cases. However, a lingering question is whether a rewards and sanctions committee is adequately capacitated and well formulated to address the more nuanced moral and ethical dilemmas and how to establish the criteria for determining what qualifies as an ethical issue or professional misconduct.

#### Core management meetings

The delivery of high-quality healthcare involves a concerted and teamwork-oriented approach, engaging both clinical and administrative staff. Management meetings are a main stay of public health institutions globally and are pivotal in directing the focus towards hospital performance, patient health, quality of care, and efficiency outcomes. They serve as a platform for creating a conducive practice environment, supporting executive management, resolving conflicts, fostering team cohesion, and facilitating continuous professional development [62–64]. Through collective thinking and deliberations, innovative solutions to clinical challenges are generated, cases discussed and action plans developed. An illustrative study highlighted how contributions from all clinic doctors in such meetings resulted in an enhanced understanding of the problem and a shared sense of well-being [65]. What remains for research to fill are gaps in the formation, frequency and the general functionality of these meetings in light of the growing burden of ethical dilemmas that require practical real-time attention.

#### Clinical department meetings

Our results showed that these meetings serve as forums for discussing a broad range of issues including patient-related matters, clinical narratives, team building, and initiatives aimed at enhancing patient quality. In a South African study, nursing unit managers through these meetings allocated 25.8% of their time to direct patient care, which involved addressing patient issues [37]. Similarly in Uganda, a fully-fledged Ministry of Health department of quality control and assurance convenes to institute measures to enhance patient safety and quality of care as per the Ministry standards [66].

### A case for establishing clinical ethics committees

Our results showed that the existing mechanisms for addressing ethical dilemmas at the UCI were insufficient and fraught with challenges. The UCI lacked policies to support clinical ethics support services such as a clinical ethics committee. There is also no evidence of support for such services in Uganda at national and hospital level, yet this support is vital for the prioritization of the existence and functionality of clinical ethics committees through allocation of budgets for these programs and ensuring protected time for individuals that provide these services. Many developed countries, including the USA, Norway, Singapore, Canada, Germany, Netherlands, and Slovakia, have not only legally mandated the formation of clinical ethics committees in every hospital but also proactively aligned their visions and goals with institutional objectives, such as enhancing patient care and satisfaction [67–71].

Our results also revealed concerns about the competence of UCI staff that provided ethics consultations in clinical ethics. In developed countries, individuals providing clinical ethics support services are trained and experienced in ethics, have sufficient knowledge, skills and character traits to address the range of ethical challenges brought to them [72, 73]. In fact, standards for assessment of core competencies and skills for clinical ethics consultation have been developed for efficiency in operations and easy pooling of experts for consultation [74, 75]. In the United States, formal apprenticeship training programs that qualify one to be a clinical ethics consultant have also been developed [76, 77]. Clinical ethics training in Uganda, however, is not as abundantly available as that of the first world countries. Although basic knowledge of ethical principles is taught in medical, nursing and pharmacy schools, it is not sufficient to meet clinical ethics challenges in the real world [78, 79].

Plans to elevate the UCI into a leading centre of oncology service delivery, training, and research in East and Central Africa are underway. The institute is already experiencing a surge in patients from various regions across Uganda and neighbouring countries, significant infrastructure investments, introduction of advanced oncology services, and increased involvement in sophisticated research. The inevitably growing complexity of clinical ethics issues at UCI necessitates robust clinical ethics consultation services.

Our results support the establishment of a multidisciplinary clinical ethics committee. This has also been supported by existing literature [80, 81]. These committees are important as they have proven effective in resource allocation, cost reduction, improved quality of care, and alleviating moral distress among HCPs [18, 82–86].

### Strengths and limitations of the study

The subjectivity of responses from qualitative questions makes it impractical to generalize the findings of the study to all hospitals in Uganda. Further explorative studies in different regions of the country are needed to understand mechanisms they utilize to resolve ethical dilemmas and recommendations of what approaches would be feasible in their context. This cross-sectional study was also limited in accounting for the entire continuum of clinical ethics consultation since data was collected at a specific point in time. Despite these limitations, this study demonstrates the need for establishment of clinical ethics support services in different hospitals in Uganda.

## Conclusion

This qualitative explorative study conducted at the UCI revealed six mechanisms for resolution of ethical dilemmas, highlighting the absence of a formally constituted and well-established clinical ethics committee. HCPs, either as individuals or teams, addressed ethical issues and dilemmas during non-ethics related meeting forums, relying on limited ethical evidence to make thoroughly thought-through decisions. These approaches were implicit, with stakeholder uncertainties about their effectiveness in resolution of ethical dilemmas. There is a need to establish a policy guided and well-supported multidisciplinary clinical ethics committee at UCI, along with provision of initial and continuous clinical ethics training of its members.

### Electronic supplementary material

Below is the link to the electronic supplementary material.

Supplementary Material 1: Interview questionnaires

Supplementary Material 2: COREQ checklist

## Data Availability

The dataset to this study was published in Harvard Dataverse with access restrictions. Access can however be granted upon request from the corresponding author.
