# Supplementary material for: Exploration of clinical ethics consultation in Uganda: a case study of Uganda Cancer Institute
Source: BMC Med Ethics. 2024 Aug 9;25:87. doi: 10.1186/s12910-024-01085-1 (PMC11312825; doi:10.1186/s12910-024-01085-1)
Supplement: Supplementary file 1 — Supplementary Material 1: Interview questionnaires [file 12910_2024_1085_MOESM1_ESM.docx]

**Qualitative interview guide for exploration of clinical ethics consultation at Uganda Cancer Institute.**

1. Tell me what you understand by the terms ethical issues and ethical dilemmas / decision making dilemmas. (Probe for a clear understanding of the ethical dilemmas)
2. With your interaction with patients/caretakers/healthcare workers, what are some of the decision-making dilemmas you have encountered during your health care.
3. What approaches did you use to resolve the ethical issue/dilemma you were faced with? Could you give me an example of a case with ethical dilemmas that particularly impressed you?
4. Describe to me some measures, guidelines or policies at the Uganda Cancer Institute through which the above dilemmas are addressed and resolved?
5. Could you please tell me some of the reasons that influenced you to seek resolutions of the challenges you faced while providing/seeking health care.
6. What factors do you think have influenced clinical ethics consultation services offered at the UCI. Please share with me as many as you can.
7. Kindly share with me your experience and perspective on the existing processes of clinical ethics consultation at the Uganda Cancer Institute? What is your experience with these approaches/CESS, in terms of effect on patient and health system outcomes, meeting their expectations, and their functionality?
8. What recommendations would you make for improved clinical ethics at the Uganda Cancer Institute?
9. What considerations would you make in establishment of a clinical ethics committee at UCI. What would be some of the challenges that medical facilities might face in the establishment of such a committee.
