# Supplementary material for: Exploration of clinical ethics consultation in Uganda: a case study of Uganda Cancer Institute
Source: BMC Med Ethics. 2024 Aug 9;25:87. doi: 10.1186/s12910-024-01085-1 (PMC11312825; doi:10.1186/s12910-024-01085-1)
Supplement: Supplementary file 2 — Supplementary Material 2: COREQ checklist [file 12910_2024_1085_MOESM2_ESM.docx]

Completed COREQ checklist

| **Domain 1: Research team and reflexivity** | | | |
| --- | --- | --- | --- |
| Personal characteristics | | | |
| 1.Interviewer/facilitator | | Which author/s conducted the interview or  focus group? | MMN carried out the interviews with the help one hired professional research assistant who took notes |
| 2.Credentials | | What were the researcher's credentials? *E.g.*  *PhD, MD* | NKS and OK each have a PhD. PK is undertaking his PhD. MMN has a MD. |
| 3.Occupation | | What was their occupation at the time of the  study? | MMN was a bioethics fellow. NKS, OK and PK were senior bioethics lecturers at Makerere University. |
| *4.*Gender | | Was the researcher male or female? | MMN and OK were female. NKS and PK were male. |
| *5.*Experience and  training | | What experience or training did the researcher  have? | NKS, PK and OK had all undertaken training in qualitative research methodologies and had previous experience of this methodology. |
| Relationship with participants | | | |
| *6.* | Relationship  established | Was a relationship established prior to study  commencement? | No prior relationship was established between the researchers and participants. |
| *7.* | Participant knowledge of the interviewer | What did the participants know about the researcher? *E.g. Personal goals, reasons for*  *doing the research* | Participants knew where the researchers worked and the purpose of the research. |
| *8.* | Interviewer characteristics | What characteristics were reported about the interviewer/facilitator? *E.g. Bias, assumptions,*  *reasons and interests in the research topic* | MMN had interest in the research. She was a bioethics fellow that previously worked at the Uganda Cancer Institute. |
| **Domain 2: Study design** | | | |
| Theoretical framework | | | |
| *9.* | Methodological orientation and theory | What methodological orientation was stated to underpin the study? *E.g. grounded theory, discourse analysis, ethnography,*  *phenomenology, content analysis* | A phenomenological approach underpinned this study. Analysis was based on a thematic framework. |
| Participant selection | | | |
| *10.* | Sampling | How were participants selected? *E.g. purposive,*  *convenience, consecutive, snowball* | Participants were purposively selected. The researchers contacted Uganda Cancer Institute (UCI) management to identify potential participants who attended for a where ethical issues and dilemmas were discussed. UCI counsellors and social workers were also contacted to identify potential patient and caretaker participants that had experienced ethical issues/dilemmas. |
| *11.* | Method of approach | How were participants approached? *E.g. face-*  *to-face, telephone, mail, email* | Potential participants were reached out to via email, SMS and phone call for the interview appointment. |
| *12.* | Sample size | How many participants were in the study? | Twenty-one in-depth interviews (twelve with UCI staff and nine with patients) and three focus group discussions (two with patients and one with caretakers). Patient focus group discussions had six participants whilst caretaker had ten. |
| *13.* | Non-participation | How many people refused to participate or  dropped out? What were the reasons for this? | Numbers of refusals were not recorded. |
| Setting | | | |
| *14.* | Setting of data  collection | Where was the data collected? *E.g. home, clinic,*  *workplace* | Focus group discussions and in-depth interviews with patients and caretakers took place in a designated room. In-depth interviews with UCI staff took place in their respective offices. |
| *15.* | Presence of non-  participants | Was anyone else present besides the  participants and researchers? | Only MMN and a professional research assistant were present. |
| *16.* | Description of sample | What are the important characteristics of the  sample? *E.g. demographic data, date* | Included in table 1, page 8 |
| Data collection | | | |
| *17.* | Interview guide | Were questions, prompts, guides provided by  the authors? Was it pilot tested? | Described in data collection methods and instruments section (Page 5) and attached as supplementary file. |
| *18.* | Repeat interviews | Were repeat interviews carried out? If yes, how  many? | No repeat in-depth and focus group interviews were carried out |
| *19.* | Audio/visual recording | Did the research use audio or visual recording  to collect the data? | Interviews and focus group sessions were audio-recorded. |
| *20.* | Field notes | Were field notes made during and/or after the  interview or focus group? | Field notes were taken by a hired professional research assistant. |
| *21.* | Duration | What was the duration of the interviews or  focus group? | Focus group sessions and interviews lasted between forty five and sixty minutes. |
| *22.* | Data saturation | Was data saturation discussed? | Described in data collection and instruments section, page 6. |
| *23.* | Transcripts returned | Were transcripts returned to participants for  comment and/or correction? | Four transcripts were returned to participants for validation. |
| **Domain 3: analysis and findings** | | | |
| Data analysis | | | |
| *24.* | Number of data  coders | How many data coders coded the data? | MMN and the hired professional research assistant independently coded the data. |
| *25.* | Description of the  coding tree | Did authors provide a description of the coding  tree? | A coding frame was developed and available upon request from the corresponding author. |
| *26.* | Derivation of themes | Were themes identified in advance or derived  from the data? | Themes were identified in advance (deductively). More themes were derived from the data (inductively). Generated themes were discussed among the authors until a consensus was reached. |
| *27.* | Software | What software, if applicable, was used to  manage the data? | Nvivo 12 |
| *28.* | Participant checking | Did participants provide feedback on the  findings? | Four participants were contacted for review and confirmation on accuracy of information transcribed. Clarification was provided on what mechanisms they used to resolve ethical issues/dilemmas and their knowledge of existing mechanism at the UCI to resolve such issues. |
| Reporting | | | |
| *29.* | Quotations presented | Were participant quotations presented to illustrate the themes / findings? Was each  quotation identified? *E.g. Participant number* | Quotations have been presented throughout the results section, with participant codes assigned to all participants and used against quotations. |
| *30.* | Data and findings  consistent | Was there consistency between the data  presented and the findings? | We endeavored to report the study findings in a clear, consistent manner in order to accurately reflect the data that have been collected. |
| *31.* | Clarity of major  themes | Were major themes clearly presented in the  findings? | Yes, main themes are clearly presented in the results section (page 9-15). |
| *32.* | Clarity of minor  themes | Is there a description of diverse cases or  discussion of minor themes? | All data relating to the development of the algorithm is presented in the results section (page 9-15) |
